# Contrast-enhanced CT radiomics for preoperative prediction of stage in epithelial ovarian cancer: a multicenter study

**DOI:** 10.1186/s12885-024-12037-8

**Published:** 2024-03-06

**Authors:** Yinping Leng, Ao Kan, Xiwen Wang, Xiaofen Li, Xuan Xiao, Yu Wang, Lan Liu, Lianggeng Gong

**Affiliations:** 1 Department of Radiology, the Second Affiliated Hospital of Nanchang University, Minde Road No. 1, 330006 Nanchang, Jiangxi Province China; 2 Department of Radiology, Jiangxi Cancer Hospital, Nanchang, Jiangxi China; 3 Department of Radiology, Jiangxi Provincial People’s Hospital, Nanchang, Jiangxi China; 4 Clinical and Technical Support, Philips Healthcare, Shanghai, China

**Keywords:** Epithelial ovarian cancer, CT, Stage, Radiomics, Machine learning

## Abstract

**Background:**

Preoperative prediction of International Federation of Gynecology and Obstetrics (FIGO) stage in patients with epithelial ovarian cancer (EOC) is crucial for determining appropriate treatment strategy. This study aimed to explore the value of contrast-enhanced CT (CECT) radiomics in predicting preoperative FIGO staging of EOC, and to validate the stability of the model through an independent external dataset.

**Methods:**

A total of 201 EOC patients from three centers, divided into a training cohort (*n* = 106), internal (*n* = 46) and external (*n* = 49) validation cohorts. The least absolute shrinkage and selection operator (LASSO) regression algorithm was used for screening radiomics features. Five machine learning algorithms, namely logistic regression, support vector machine, random forest, light gradient boosting machine (LightGBM), and decision tree, were utilized in developing the radiomics model. The optimal performing algorithm was selected to establish the radiomics model, clinical model, and the combined model. The diagnostic performances of the models were evaluated through receiver operating characteristic analysis, and the comparison of the area under curves (AUCs) were conducted using the Delong test or F-test.

**Results:**

Seven optimal radiomics features were retained by the LASSO algorithm. The five radiomics models demonstrate that the LightGBM model exhibits notable prediction efficiency and robustness, as evidenced by AUCs of 0.83 in the training cohort, 0.80 in the internal validation cohort, and 0.68 in the external validation cohort. The multivariate logistic regression analysis indicated that carcinoma antigen 125 and tumor location were identified as independent predictors for the FIGO staging of EOC. The combined model exhibited best diagnostic efficiency, with AUCs of 0.95 in the training cohort, 0.83 in the internal validation cohort, and 0.79 in the external validation cohort. The F-test indicated that the combined model exhibited a significantly superior AUC value compared to the radiomics model in the training cohort (*P* < 0.001).

**Conclusions:**

The combined model integrating clinical characteristics and radiomics features shows potential as a non-invasive adjunctive diagnostic modality for preoperative evaluation of the FIGO staging status of EOC, thereby facilitating clinical decision-making and enhancing patient outcomes.

**Supplementary Information:**

The online version contains supplementary material available at 10.1186/s12885-024-12037-8.

## Background

Epithelial ovarian cancer (EOC) is the most prevalent malignant neoplasm of the ovaries, with the highest fatality rate among gynecological malignancies [1, 2]. Despite significant progress achieved in chemotherapy regimens and targeted therapy, the prognosis remains unsatisfactory, with a 5-year survival rate of less than 40% [3]. According to the International Federation of Gynecology and Obstetrics (FIGO) staging system, EOC patients are classified as early-stage (FIGO stage I-II) or advanced-stage (FIGO stage III-IV) [4]. The treatment options for EOC patients largely depend on the FIGO stage at the time of diagnosis. Fertility-sparing surgery is a viable option for patients with early-stage EOC, potentially avoiding chemotherapy [5]. However, in advanced-stage EOC, when initial cytoreductive surgery is unlikely to achieve complete tumor shrinkage, combination neoadjuvant chemotherapy should be considered [6]. Accurate staging is crucial for predicting the prognosis of EOC patients and determining appropriate treatment plans [7]. However, achieving accurate FIGO staging typically requires invasive surgery or tissue biopsy, which comes with potential risks such as disease metastasis [8].

Various imaging modalities, including positron emission tomography (PET)/CT, CT, and magnetic resonance imaging (MRI), have been explored to assess feasibility of tumor resection, eligibility for efficacious cytoreductive surgery, and the necessity of postoperative chemotherapy in cases where optimal tumor reduction is not achieved [9, 10]. The diagnostic effectiveness of PET/CT in EOC is comparatively inferior to that of MRI and CT, exhibiting lower sensitivity and specificity [11]. Despite its high soft tissue contrast MRI does not significantly outperform CT in detecting peritoneal implantation metastasis in late-stage EOC patients [12]. CT scanning, a rapid and widely used imaging technique, can be utilized for stratified treatment of EOC and to assess the initial extent of the disease for surgical planning purposes [13]. The European Society of Urogenital Radiology recommends CT as the preferred imaging method for preoperative staging and follow-up of EOC [7]. Nevertheless, the non-invasive CT approach for assessing the FIGO staging of EOC predominantly relies on the subjective expertise of radiologists.

Radiomics involves the high-throughput extraction of numerous latent image features from CT or MRI images, coupled with modeling and analytical techniques such as machine learning, to transform digital medical images into multidimensional data that can be explored [14]. EOC is known for its tumor heterogeneity, which manifests as spatial variations at the morphological and histopathological levels, encompassing alterations in cell count, angiogenesis, necrosis, and extracellular matrix [15, 16]. Radiomics has emerged as a significant non-invasive modality for assessing the heterogeneity of malignant tumors. This technique has the ability to identify subtle structures and uncover potential image information that may not be discernible to the unaided eye, thereby enhancing the accuracy of diagnosis and prognosis evaluation [17, 18]. Recent investigations have explored the utility of CT-based radiomics in differential diagnosis, treatment prognosis, recurrence risk, and assessment of tumor heterogeneity [19–22]. Thus, it is posited that a CT radiomics model predicated on primary tumors of EOC may yield precise prognostication of FIGO staging. The objective of our study is to evaluate the performance of a contrast-enhanced CT (CECT) radiomics model in predicting the FIGO staging of EOC, with the ultimate aim of assisting clinical practitioners in devising individualized treatment regimens for EOC patients.

## Methods

### Patients

**Fig. 1:**
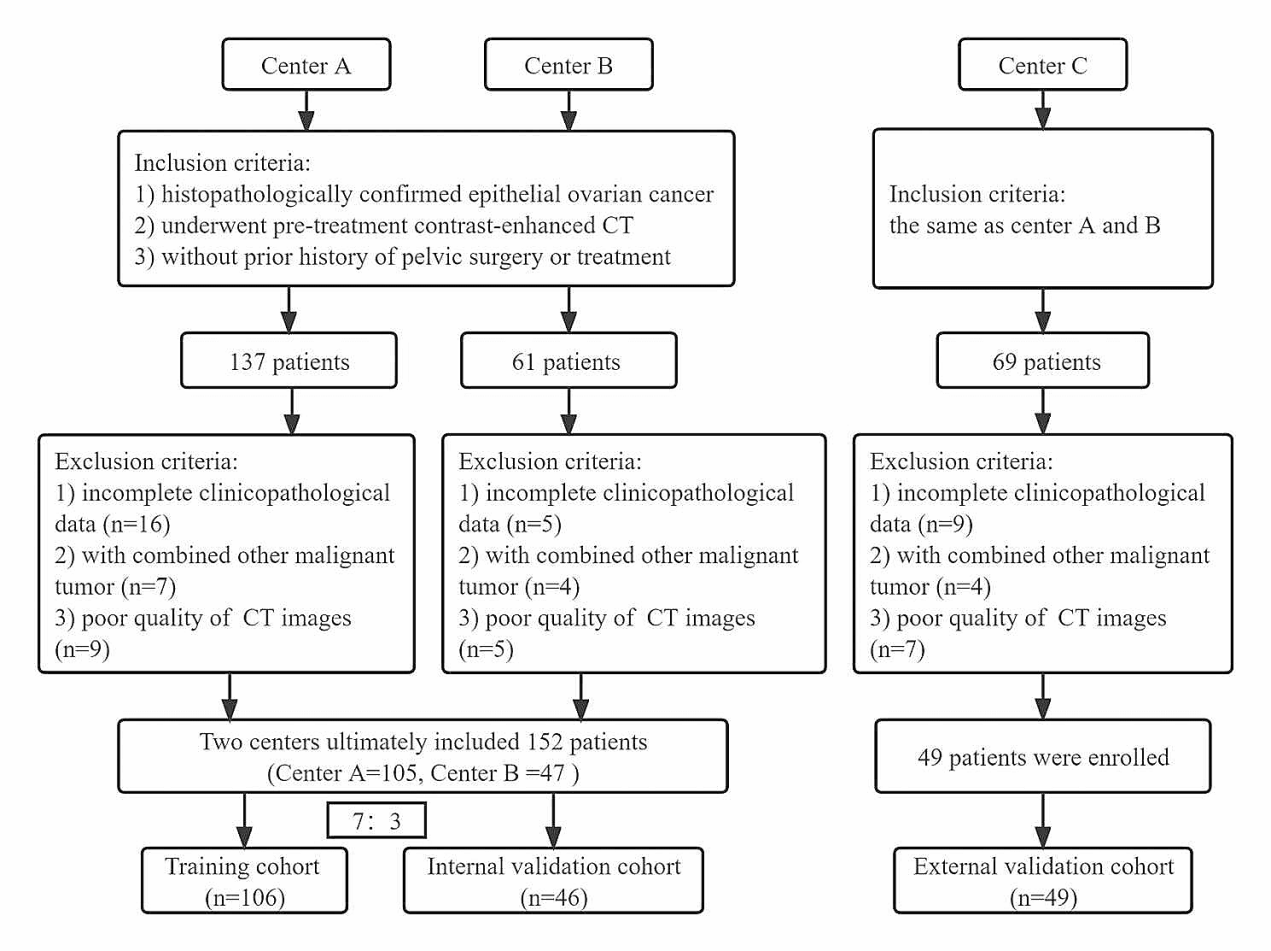
The flowchart of patient selection from three centers

This multicenter retrospective study received approval from the Institutional Review Board of three medical centers. Written informed consent for participants was not required for this study in accordance with the national legislation and the institutional requirements. The study involved a retrospective analysis of clinical data and CECT images obtained from patients diagnosed by pathology with EOC at Center A (from April 2017 to June 2022), Center B (January 2019 to September 2022), and Center C (December 2019 to October 2022). Information for these three medical centers is shown in the Additional file 1: Table S1. The inclusion criteria consisted of patients who met the following conditions: [1] histopathologically confirmed EOC, [2] no previous history of pelvic surgery or treatment, and [3] underwent pre-treatment CECT. Patients with concurrent malignant tumors, with poor CT quality, or incomplete clinicopathological records were excluded from the study. Finally, a total of 201 EOC patients were included (105 from Center A, 47 from Center B, and 49 from Center C). The flowchart of patient selection from three centers was shown in Fig. 1. According to the FIGO 2014 staging system, all patients were categorized into either the early-stage (the negative class) and advanced-stage (the positive class) groups. Patients from Center A and B were randomly divided into training and internal validation cohort in a ratio of 7:3. Data from Center C were exclusively for an external validation cohort to evaluate the generalizability of the models developed on data from different institutions.

The clinicopathological information, including preoperative carcinoma antigen 125 (CA125) and carbohydrate antigen 199 (CA199) level, age, FIGO stage, histologic subtypes, tumor location, peritoneal metastasis, Ki-67 expression, and menopausal status, was retrieved from the patients’ medical records.

### CECT images Acquisition

All patients underwent abdominopelvic CECT scans before surgery. The scans were performed using the Brilliance 16 and IQon Spectral scanners (both Philips Healthcare, Netherlands), SOMATOM scanner (Siemens Healthcare, Germany) or Discovery 750 HD scanner (GE Healthcare, USA). Detailed information regarding the CT equipment used at each center is presented in Additional file 1: Table S1. In all patients, the CECT was acquired 50 s after an injection of iodinated contrast media (370 mg I/mL, Heng Rui Pharma, Jiangsu, China; 3–4 mL/s, 1.5 mL/kg body weight) via the antecubital vein using a high-pressure syringe (Ulrich, Germany).

### Tumor segmentation and radiomic feature extraction

Tumors segmentation was performed using 3D Slicer (version 4.13.0, www.slicer.org). Firstly, a highly experienced radiologist (Radiologist A) with a decade of experience in diagnosing pelvic imaging manually delineated the volume of interest (VOI) layer by layer along the lesion’s edge, making efforts to avoid blood vessels and calcified areas. If the tumor was multifocal, only the one with the largest diameter on the axial image was delineated. To assess the reproducibility of the segmentation, another experienced radiologist (Radiologist B) with over eight years of experience in imaging diagnosis randomly selected 30 patients and replicated the segmentation using the identical methodology. After a month, Radiologist A repeated the segmentation for the same 30 patients. The Intraclass correlation coefficient (ICC) was utilized to evaluate the consistency of the CT radiomics features extracted by two radiologists from the segmentation results of the 30 patients.

Before radiomics feature extraction, resampling and intensity normalization was performed to eliminate the heterogeneity of CT scan parameters. All CECT images were preprocessed by algorithm written in Python 3.9, which resampled the images to a voxel size of 1.0 × 1.0 × 1.0 mm³ using linear interpolation. To facilitate feature extraction, the images were discretized with a bin width of 25 HU. Radiomics features were automatically extracted from the VOI using the open-source package PyRadiomics (version 3.0.1). A total of 851 radiomics features were extracted from each VOI, including 107 original features and 744 wavelet features. These features encompassed various categories, including first-order features (*n* = 162), shape features (*n* = 14), gray-level run length matrix (GLRLM) features (*n* = 144), gray-level dependency matrix (GLDM) features (*n* = 126), gray-level co-occurrence matrix (GLCM) features (*n* = 216), neighborhood gray-tone difference matrix (NGTDM) features (*n* = 45), and gray-level size zone matrix (GLSZM) features (*n* = 144).

### Feature postprocessing and feature selection

To reduce the dimensionality of radiomics features, we employed a three-step approach to select the features within the training cohort. Initially, the radiomics features of the training cohort were standardized through Z-score normalization. Subsequently, the maximum relevance minimum redundancy (mRMR) and spearman’s rank correlation coefficient method were utilised to retain the best radiomics features for the prediction and eliminate the irrelevant and redundant ones. Finally, the least absolute shrinkage and selection operator (LASSO) algorithm was utilized to optimize the regularization parameter (λ) by employing to the 1-standard error of the minimum. This approach effectively controls the level of regularization, thereby reducing the complexity of the radiomics model by incorporating selected features with non-zero coefficients.

### Radiomics models building

We utilized five different machine learning algorithms, including regularized logistic regression (LR), support vector machine (SVM), random forest (RF), light gradient boosting machine (LightGBM), and decision tree (DT), to construct the radiomics models. We used 10-fold cross-validation for hyper-parameter tuning. The optimal machine learning algorithm was selected based on the evaluation of both fitting performance and generalization performance.

### Combined model building and evaluation

**Fig. 2:**
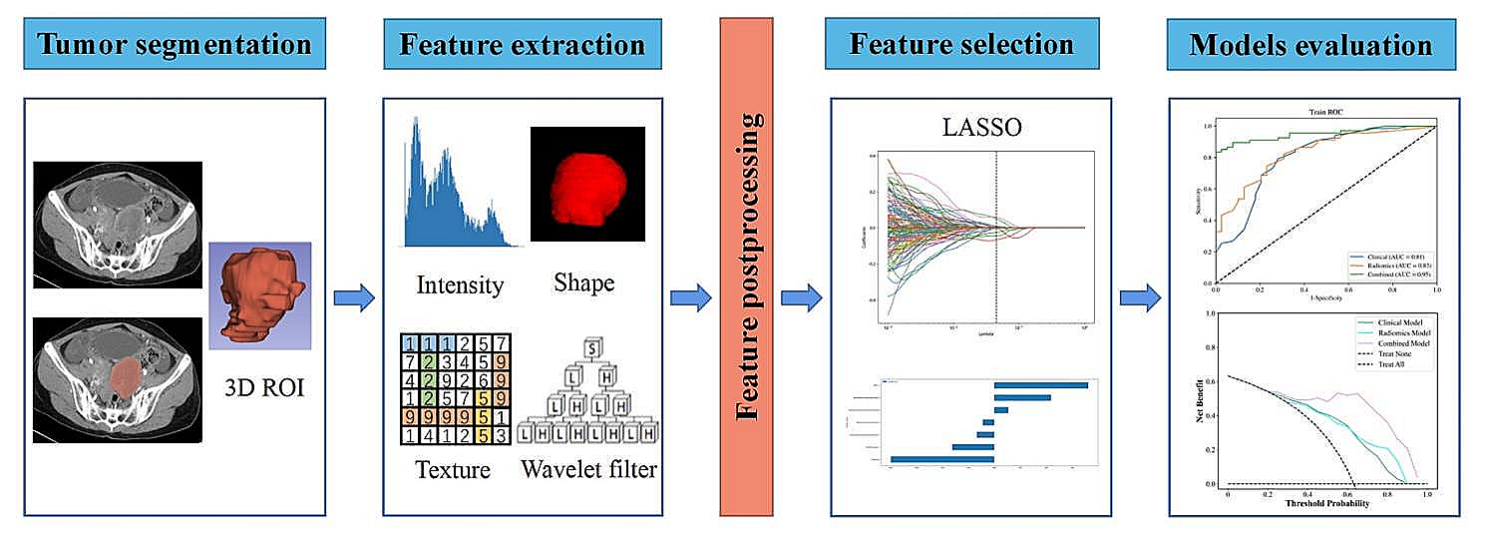
The workflow of radiomics. LASSO, least absolute shrinkage and selection operator

The clinical characteristics included preoperative CA125 and CA199 level, age, tumor location, and menopause status. After univariate logistic regression analyses, the significant clinical characteristics variables of the training cohort were included in a multivariate logistic regression analysis to identify independent predictive factors. A clinical model was constructed by these independent predictive factors. Subsequently, a combined model was established by integrating independent predictive factors with radiomics features (λ ≠ 0). The aim was to investigate whether this integration could further improve the predictive performance. To ensure comparability, we employed the optimal machine learning algorithm as used in the radiomics model. The overall workflow is depicted in Fig. 2, illustrating the step-by-step process of our study. The performance of the three models (clinical model, radiomics model, and combined model) in predicting advanced-stage EOC was assessed using the receiver operating characteristic (ROC) curves. The evaluation indicators for model performance include accuracy, sensitivity, specificity, and F1 score. Delong test was employed to compare the area under curve (AUC) among the clinical and radiomics models. The F-test was used to compare the AUC values of radiomics and combined models, as well as clinical and combined models. Decision curve analysis (DCA) was utilized to assess and compare the net benefit difference among the clinical model, radiomics model and the combined model across various threshold probabilities. Net benefit is a weighted composite of true and false positives, with weights derived from the threshold probability [23].

### Statistical analysis

Statistical analysis was conducted using SPSS 26.0, Python (version 3.9), and R software (version 4.2.1). The chi-square test was employed to compare qualitative data. The Kolmogorov-Smirnov test was utilized to assess the normal distribution of quantitative data. For normally distributed quantitative data, mean ± SD was used to express the results, and the t-test was employed for group comparisons. Non-normally distributed quantitative data were presented as medians with interquartile ranges, and comparisons were perfomed using the Mann-Whitney U-test. A significance level of *P* < 0.05 was considered statistically significant. The R packages and Python libraries used in this study are provided in **Additional file 2: S1**.

## Results

### Clinical characteristics

A total of 201 patients (77 in the early-stage group and 124 in the advanced-stage group) were included in this study, with a training cohort of 106, an internal validation cohort of 46, and an external validation cohort of 49. Details of the clinicopathological characteristics between the two groups are listed in Table 1. Significant differences were observed between the groups in CA125, histologic subtypes, tumor location and peritoneal metastasis (*P* < 0.05). There was a statistically significant difference in CA125 levels and tumor location between the early-stage and advanced-stage groups within the training cohort (*P* < 0.05) (Table 2). No significant differences were seen in terms of clinical characteristics among these three data cohorts (all *P* > 0.05). The results of the multivariate logistic regression analysis indicated that CA125 [*P* = 0.005, odds ratio (OR) = 1, confidence interval (CI) = 1-1.001] and tumor location (*P* = 0.027, OR = 2.228, CI = 1.094–4.538) were identified as significant independent predictors for the FIGO staging of EOC.

**Table 1:** The clinicopathological and radiological characteristics of EOC patients

Characteristics	Early-stage (*n* = 77)	Advanced-stage (*n* = 124)	P-value
Age (years)	53 (49, 59.5)	56 (49, 65)	0.144^1^
CA125 (U/mL)	229.4 (98.16, 614.5)	815.95 (249.73, 2187)	< 0.001^1^
CA199 (U/mL)	12.95 (5.4, 43.83)	13.03 (7.52, 26.28)	0.858^1^
Menopause status			0.721^2^
Yes	56 (72.7%)	93 (75%)	
No	21 (27.3%)	31 (25%)	
Histologic subtypes			< 0.001^2^
HGSC	45 (58.4%)	116 (93.5%)	
Non-HGSC	32 (41.6%)	8 (6.5%)	
Ki-67 expression			0.350^2^
≤ 50	40 (51.9%)	56 (45.2%)	
> 50	37 (48.1%)	68 (54.8%)	
Tumor location			< 0.001^2^
Bilateral	26 (33.8%)	80 (64.5%)	
Unilateral	51 (66.2%)	44 (35.5%)	
Peritoneal metastasis			< 0.001^2^
Yes	8 (10.4%)	94 (75.8%)	
No	69 (89.6%)	30 (24.2%)	

Data are presented as median (interquartile range) for non-normally distributed continuous variables, or number (%) for categorical variables. EOC, epithelial ovarian cancer; CA125, carcinoma antigen 125; CA199, carbohydrate antigen 199; HGSC, high-grade serous carcinoma. ^1^Mann-Whitney U test. ^2^chi-square test

**Table 2:** Clinical characteristics of the training and validation cohorts

Characteristics	Training (*n* = 106)		P-value	Internal validation (*n* = 46)		P-value	External validation (*n* = 49)		P-value
	Early-stage (*n* = 39)	Advanced- stage (*n* = 67)		Early-stage (*n* = 17)	Advanced- stage (*n* = 29)		Early-stage (*n* = 21)	Advanced- stage (*n* = 28)	
Age (years)	54.56 ± 7.76	55.76 ± 9.72	0.513^1^	53.1 ± 13.6	58.31 ± 10.29	0.146^1^	55.57 ± 7.97	56.07 ± 8.67	0.837^1^
Menopause			0.690^2^			0.276^2^			0.930^2^
Yes	31 (79.5%)	51 (76.1%)		11 (64.7%)	23 (79.3%)		14 (66.7%)	19 (67.9%)	
No	8 (20.5%)	16 (12.9%)		6 (35.3%)	6 (20.7%)		7 (33.3%)	9 (32.1%)	
CA125 (U/mL)	236.2 (120.4, 630)	1158 (247, 2973.7)	0.002^3^	221.6 (88, 622.2)	676 (255,1187.3)	0.033^3^	224.9 (73.0, 802)	1058 (332.7,1997)	0.003^3^
CA199 (U/mL)	14.38 (5.75, 38.2)	14.7 (8.9, 27.6)	0.593^3^	9.33 (4.0, 36.3)	14.58 (8.11, 28.7)	0.937^3^	13.51 (6.1, 149.9)	9.52 (5.48, 19.3)	0.332^3^
Tumor location			0.027^2^			0.079^2^			< 0.001^2^
Bilateral	14 (35.9%)	39 (58.2%)		6 (35.3%)	18 (62.1%)		6 (28.6%)	23 (82.1%)	
Unilateral	25 (64.1%)	28 (41.8%)		11 (64.7%)	11 (37.9%)		15 (71.4%)	5 (17.9%)	

Data are presented as mean ± standard deviation for normally distributed continuous variables, median (interquartile range) for non-normally distributed continuous variables, or number (%) for categorical variables. CA125, carcinoma antigen 125; CA199, Carbohydrate antigen 199. ^1^t test. ^2^chi-square test. ^3^Mann-Whitney U test

### Feature selection

**Fig. 3:**
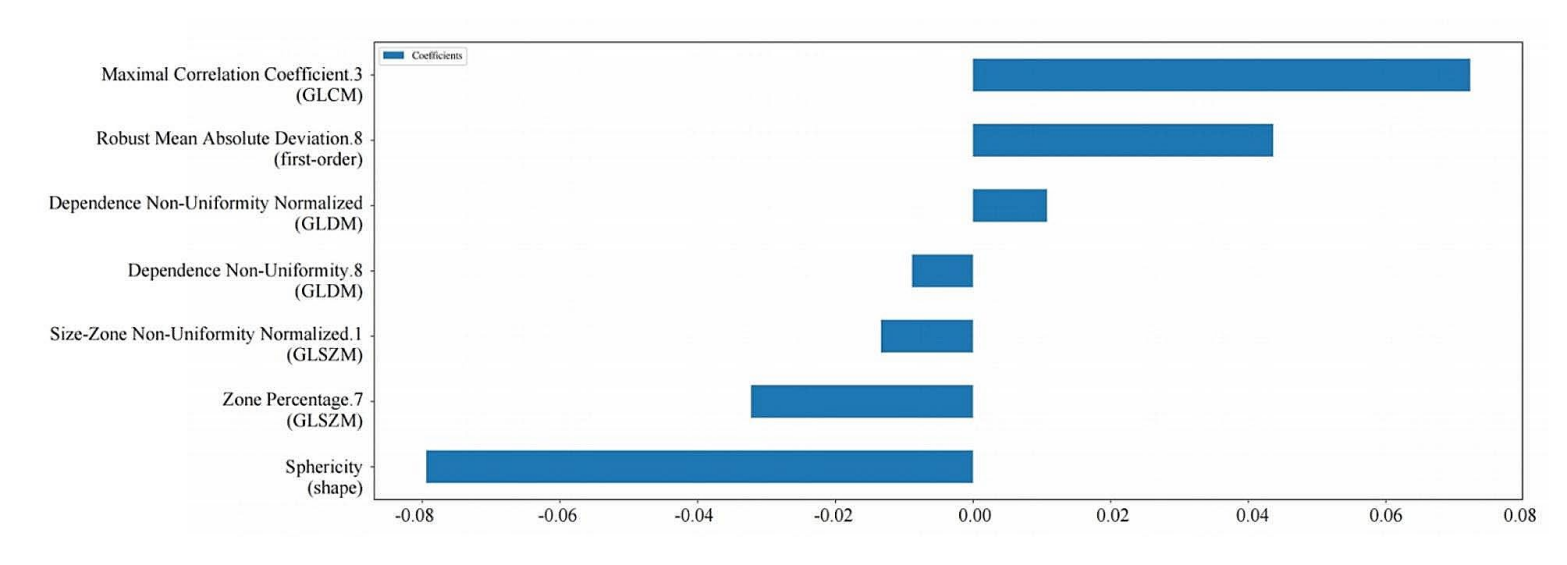
Radiomics features selected by the least absolute shrinkage and selection operation regression method and their corresponding weights. GLCM, gray-level co-occurrence matrix; GLDM, gray-level dependency matrix; GLSZM, gray-level size zone matrix

A total of 851 radiomics features were initially extracted from each CT image. Subsequently, 610 radiomics features demonstrating good consistency (ICC > 0.75) were selected for further feature screening. Then, the mRMR and spearman’s rank correlation coefficient method were used to retain 30 optimal radiomics features for predicting FIGO staging. Finally, the LASSO algorithm identified seven features with non-zero coefficients in the training cohort which were used to construct the radiomics model (Additional file 3: Figure S1). The weight of each feature was depicted in Fig. 3.

### Radiomic models building and comparison

The results of constructing radiomics models using five machine learning algorithms are presented in Table 3. The LR model outperformed the SVM model in the training cohort (AUC of 0.80 vs. 0.75). However, both the SVM model and LR model showed unstable in the external validation cohort, with low AUCs (AUC of 0.54 and 0.51 respectively). Conversely, the DT, and RF models exhibited trend of overfitting across all three cohorts. The LightGBM model displayed promising AUCs of 0.83, 0.80, and 0.68 in the training cohort, internal validation cohort, and external validation cohort, respectively. These results indicate that the radiomics model constructed using the LightGBM algorithm emerges as the optimal choice for preoperative FIGO staging prediction of EOC.

**Table 3:** The results of five machine learning algorithms utilized for constructing radiomics models

Model	AUC (95% CI)	Sensitivity	Specificity	Accuracy	F1 score
LightGBM					
Training	0.83 (0.75–0.91)	0.75	0.77	0.75	0.79
Internal validation	0.80 (0.67–0.93)	0.76	0.65	0.72	0.77
External validation	0.68 (0.52–0.84)	0.68	0.76	0.71	0.73
LR					
Training	0.80 (0.71–0.89)	0.75	0.77	0.76	0.82
Internal validation	0.80 (0.66–0.93)	0.72	0.77	0.74	0.78
External validation	0.51 (0.34–0.67)	0.46	0.48	0.47	0.40
SVM					
Training	0.75 (0.65–0.86)	0.88	0.59	0.77	0.81
Internal validation	0.83 (0.71–0.96)	0.72	0.88	0.78	0.81
External validation	0.54 (0.37–0.70)	0.64	0.38	0.53	0.44
DT					
Training	0.95 (0.91–0.98)	0.75	1.00	0.84	0.89
Internal validation	0.62 (0.45–0.79)	0.97	0.41	0.76	0.84
External validation	0.60 (0.44–0.75)	0.86	0.33	0.63	0.73
RF					
Training	0.99 (0.98-1.00)	0.93	1.00	0.95	0.95
Internal validation	0.75 (0.60–0.90)	0.76	0.71	0.74	0.78
External validation	0.67 (0.51–0.83)	0.54	0.81	0.65	0.69

LightGBM, light gradient boosting machine; LR, logistic regression; SVM, support vector machine; DT, decision tree; RF, random forest; AUC, area under the curve; 95% CI, 95% confidence interval

### Combined model evaluation

**Fig. 4:**
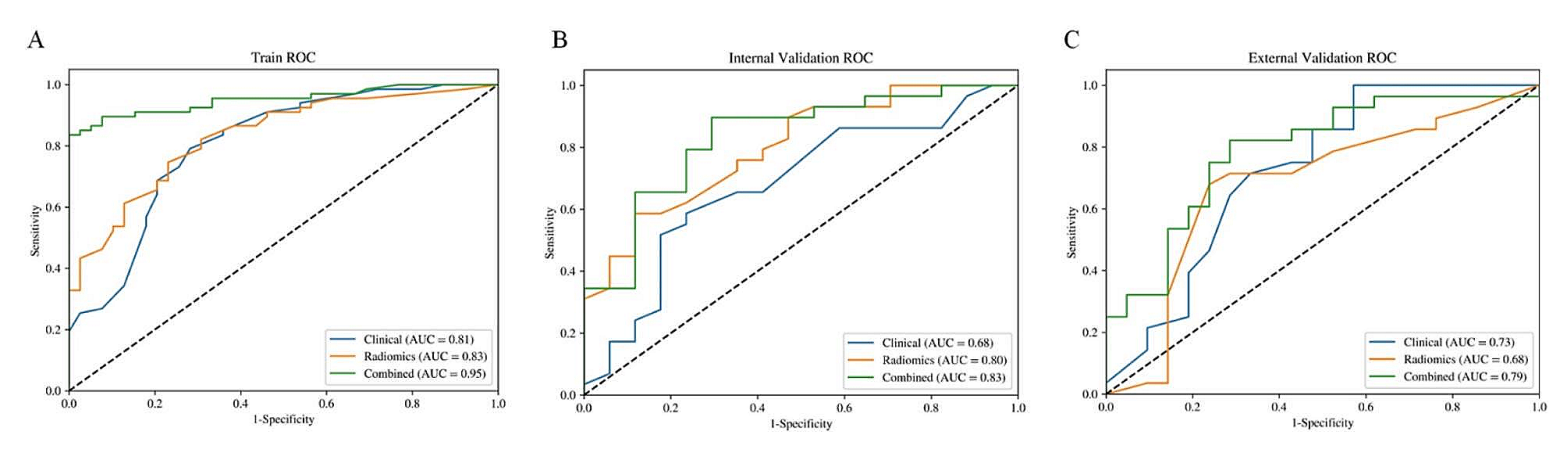
Receiver operating characteristic curves for the clinical model, radiomics model, and combined model in the training cohort (A), internal validation cohort (B), and the external validation cohort (C). ROC, receiver operating characteristic. AUC, area under the curve

**Fig. 5:**
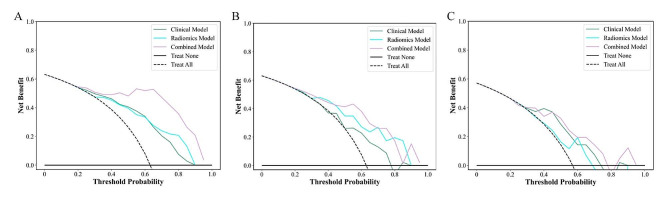
Decision curve analysis of clinical model, radiomics model, and combined model in the training cohort (A), internal validation cohort (B), and external validation cohort (C). The combined model had a higher overall net benefit in the differentiation of advanced-stage (dashed line) from early-stage (black solid line) across the full range of threshold probabilities at which an EOC patient would have an FIGO stage-positive

Table 4 lists the performance metrics of the three models for prediction EOC stage. Figure 4 shows the ROC curves of the three models for the training, internal validation and external validation cohorts. The combined model demonstrated the highest diagnostic efficiency, achieving the AUCs of 0.95 (95% CI, 0.91–0.99; accuracy, 90%; sensitivity, 84%; specificity, 100%; F1 score, 91%) in training cohort, 0.83 (95% CI, 0.71–0.96; accuracy, 83%; sensitivity, 90%; specificity, 71%; F1 score, 87%) in the internal validation cohort, and 0.79 (95% CI, 0.66–0.92; accuracy, 78%; sensitivity, 82%; specificity, 71%; F1 score, 81%) in the external validation cohort. In the internal validation cohort, the radiomics model achieved a moderate AUC of 0.80 (95% CI, 0.67–0.93), outperforming the clinical model with an AUC of 0.68 (95% CI, 0.51–0.85). The Delong test showed that there were no differences in AUC values between the clinical model and the radiomics model among the three cohorts (all *P* > 0.05). F-test showed that the AUC values of the combined model were significantly better than those of the radiomics model in the training cohort, internal validation cohort, and external validation cohort (*P* < 0.001, *P* = 0.024, *P* = 0.011, respectively). However, the AUC value of the combined model was only higher than that of the clinical model in the training cohort (*P* = 0.016). The DCA indicated that the combined model had a higher total benefit than the clinical model and radiomics model across the most reasonable threshold probabilities, meaning that the combined model was useful in predicting FIGO stage status in EOC patients (Fig. 5).

**Table 4:** Predictive performance of the three models for staging EOC.

Model	AUC (95% CI)	Sensitivity	Specificity	Accuracy	F1 score
Training cohort					
Clinical model	0.81 (0.72–0.90)	0.79	0.72	0.76	0.81
Radiomics model	0.83 (0.75–0.91)	0.75	0.77	0.75	0.79
Combined model	0.95 (0.91–0.99)	0.84	0.99	0.90	0.91
Internal validation cohort					
Clinical model	0.68 (0.51–0.85)	0.59	0.76	0.65	0.68
Radiomics model	0.80 (0.67–0.93)	0.76	0.65	0.72	0.77
Combined model	0.83 (0.71–0.96)	0.90	0.71	0.83	0.87
External validation cohort					
Clinical model	0.73 (0.57–0.88)	0.71	0.67	0.69	0.73
Radiomics model	0.68 (0.52–0.84)	0.68	0.76	0.71	0.73
Combined model	0.79 (0.66–0.92)	0.82	0.71	0.78	0.81

EOC, epithelial ovarian cancer; AUC, area under the curve; 95% CI, 95% confidence interval

## Discussion

In this multicenter study, a combined model was developed that integrates radiomics features and clinical characteristics to forecast the FIGO stage of patients with EOC. The combined model exhibited superior predictive efficacy in staging EOC, surpassing both the individual clinical model and radiomics model. External independent datasets were employed to validate the model, affirming its predictive worth and generalizability, while also highlighting its potential for clinical application. Our study suggests that the combined model has the potential to be a noninvasive tool for staging EOC, which could assist in the decision-making process for selecting a therapeutic strategy.

CA125 is presently the most frequently employed biomarker for ovarian cancer, with over 80% of EOC patients showing elevated blood levels of this marker. CA125 serves as a crucial reference indicator for assessing the effectiveness of diagnostic and treatment approaches, as well as predicting the likelihood of recurrence in EOC [24]. However, it is important to note that CA125 lacks specificity as a diagnostic marker for EOC, as it also exhibits a certain degree of positivity in other malignancies such as lung cancer and various gynecological tumors. The American College of Radiology appropriateness criteria guidelines have assigned CA125 with a rating of utmost appropriateness, comparable to CT, underscoring the comprehensive significance of CA125 in preoperative staging [25]. The present study has revealed that both tumor location and CA125 are independent risk factors for diagnosing FIGO staging of EOC patients. After being incorporated into the clinical model, the AUC value was 0.81 (95% CI, 0.72–0.90), while the sensitivity and accuracy were 79% and 76% respectively. The diagnostic performance in predicting the FIGO staging of EOC was deemed to be moderate.

Radiomics is capable of extracting a substantial quantity of quantitative imaging characteristics that are indicative of texture, intensity, heterogeneity, and morphological information with a high degree of efficiency. These characteristics are not discernible through visual assessment, but they can reflect tumor heterogeneity at the cellular level [19, 26]. In our radiomics model, the feature with the highest weightage is shape sphericity. The concept of sphericity relates to the degree of circularity of a shape in comparison to a sphere [27]. Our observations reveal a statistically significant decrease in sphericity among patients with advanced-stage EOC compared to those in early-stage. This phenomenon may be attributed to the distinct growth patterns exhibited by the two stages. Early-stage EOC is distinguished by a gradual and localized progression limited to the ovary. Conversely, advanced-stage EOC exhibits a significantly aggressive and genetically unstable phenotype, leading to an irregular growth pattern that deviates from a spherical shape [28]. Radiomics has the capability to forecast the FIGO staging status of EOC by analyzing the microstructural alterations in the tumor region.

Currently, diverse machine learning algorithms, including LR, RF, and SVM, have been employed in the radiomics analysis. LightGBM is an innovative and sophisticated gradient boosting decision tree algorithm that facilitates efficient parallel training. This algorithm significantly improves the speed and precision of training and inference through effective histogram optimization and acceleration techniques [29]. Dong et al. investigated whether patients with advanced non-small cell lung cancer had oligomyosis by combining chest CT radiomics features with the LightGBM classifier. After bayesian hyperparameter tuning of the LightGBM model, the model achieved better predictive performance [30]. In our study, the LightGBM model achieved a moderate AUC of 0.83, and 0.80 in the training cohort, and internal validation cohort, respectively. F1 score was employed to assess the balance between precision and recall, yielded values of 0.79 and 0.77 on the training cohort and internal validation cohort, respectively. These findings suggest that the predictive capacity of the model for FIGO staging in EOC patients is commendable, and may facilitate the development of tailored therapeutic strategies in clinical settings.

In recent years, several studies have been conducted on CT radiomics to forecast the clinicopathological attributes of ovarian cancer. These studies encompass the differentiation between primary and secondary ovarian cancers, the prediction of histologic subtypes, and the identification of lymph node metastasis associated with ovarian cancer [31–33]. However, there is currently a lack of research on models for preoperative prediction of the staging of EOC. Accurately predicting the FIGO staging of EOC before treatment is crucial in formulating effective treatment strategies. Based on this, this study constructed a radiomics model by extracting high-throughput information from the CECT images. The AUC value was 0.83 (95% CI, 0.75–0.91), and the sensitivity and accuracy were 75% and 75%, respectively. The diagnostic performance of predicting the EOC staging was moderate. After incorporating the clinical variables (CA125 and tumor location) to construct a combined model, the AUC value increased to 0.95 (95% CI, 0.91–0.99), further improving the diagnostic efficacy of predicting the EOC staging. The utilization of an external independent dataset to validate the model yielded an AUC value of 0.79 (95% CI, 0.66–0.92), indicating the model’s robustness and potential for practical implementation in clinical settings. The DCA also showed that whether it is the training cohort, internal validation cohort, or external validation cohort, the combined model yielded more net benefits than other models for preoperative prediction of EOC stage. DCA helps determine which patients should receive treatment by calculating net benefits. For advanced-stage patients, the approach involves the combination of neoadjuvant chemotherapy with surgical treatment, ensuring precise and appropriate clinical interventions. Conversely, early-stage patients undergo surgical treatment alone, thus avoiding unnecessary chemotherapy.

The majority of CT radiomics studies currently are conducted by single institution, resulting in limited validation of their conclusions. Furthermore, variations in research locations, suppliers, or protocols may impact the voxel intensity spectrum, thereby compromising the universality of the model [34]. To address this, our study adopted a multicenter approach, leveraging data from two prominent institutions for the training and internal validation datasets. Additionally, an independent external dataset from another center was used to validate the model’s performance. The research findings demonstrate that the AUC of the combined model in the external validation cohort is 0.79 (95% CI, 0.66–0.92), which suggests its predictive efficacy and potential for generalization.

This multicenter study has some limitations. First, the tumor segmentation process was performed manually, potentially being influenced by the radiologist’s level of expertise. One of our future research directions will involve the exploration of automatic segmentation techniques for tumor lesions. Additionally, despite the utilization of patients from three prominent medical centers, our study’s sample size remained relatively small. Studies with larger sample sizes are needed to further validate our findings in subsequent investigation.

## Conclusions

In summary, the combined model based on CECT radiomics features and clinical characteristics shows promise as a non-invasive supplementary diagnostic tool for preoperative evaluation the FIGO staging status of EOC. Our discoveries could provide valuable direction for preoperative clinical decision-making and enhance patient outcomes.

### Abbreviations

EOC Epithelial ovarian cancer.

FIGO International Federation of Gynecology and Obstetrics.

PET Positron emission tomography.

MRI Magnetic resonance imaging.

CECT Contrast-enhanced computed tomography.

CA125 Carcinoma antigen 125.

CA199 Carbohydrate antigen 199.

VOI Volume of interest.

ICC Intraclass correlation coefficient.

GLRLM Gray-level run length matrix.

GLDM Gray-level dependence matrix.

GLCM Gray-level co-occurrence matrix.

NGTDM Neighborhood gray-tone difference matrix.

GLSZM Gray-level size zone matrix.

mRMR Maximum relevance minimum redundancy.

LASSO Least absolute shrinkage and selection operator.

LR Logistic regression.

SVM Support vector machine.

RF Random forest.

LightGBM Light gradient boosting machine.

DT Decision tree.

ROC Receiver operating characteristic.

AUC Area under curve.

DCA Decision curve analysis.

OR Odds ratio.

CI Confidence interval.

HGSC High-grade serous carcinoma.

### Electronic supplementary material

Below is the link to the electronic supplementary material.

Supplementary Material 1

Supplementary Material 2

Supplementary Material 3

## Data Availability

The datasets generated and/or analysed during the current study are not publicly available (because it involves patient privacy information in our country) but are available from the corresponding author on reasonable request.
