# Supplementary material for: Contrast-enhanced CT radiomics for preoperative prediction of stage in epithelial ovarian cancer: a multicenter study
Source: BMC Cancer. 2024 Mar 6;24:307. doi: 10.1186/s12885-024-12037-8 (PMC10916071; doi:10.1186/s12885-024-12037-8)
Supplement: Supplementary file 1 — Supplementary Material 1 [file 12885_2024_12037_MOESM1_ESM.docx]

**Table S1.** CT protocols of three centers.

| Parameters | Center A | | | Center B | | Center C | |
| --- | --- | --- | --- | --- | --- | --- | --- |
|  | the Second Affiliated Hospital of Nanchang University | | | Jiangxi Provincial People's Hospital | | Jiangxi Cancer Hospital | |
| CT version | Brilliance 16  (Philips Healthcare, Netherlands) | IQon  (Philips Healthcare, Netherlands) | SOMATOM  (Siemens Healthcare, Germany) | SOMATOM (Siemens Healthcare, Germany) | Discovery 750  (GE Healthcare,  USA) | Brilliance 16 (Philips Healthcare, Netherlands) | SOMATOM (Siemens Healthcare, Germany) |
| Number | 52 | 10 | 43 | 23 | 24 | 25 | 24 |
| Tube voltage (kVp) | 120 | 120 | 120 | 120 | 120 | 120 | 120 |
| Tube current  (mAs) | 260 | 250 | 200 | 200 | 220 | 260 | 200 |
| Rotation time  (s) | 0.4 | 0.5 | 0.5 | 0.5 | 0.6 | 0.4 | 0.5 |
| Image matrix | 512×512 | 512×512 | 512×512 | 512×512 | 512×512 | 512×512 | 512×512 |
| Slice thickness (mm) | 5 | 1.25 | 5 | 5 | 5 | 5 | 5 |
| Slice spacing  (mm) | 5 | 1.25 | 5 | 5 | 5 | 5 | 5 |
| Detector collimation  (mm) | 64×0.625 | 64×0.625 | 128×0.6 | 128×0.6 | 64×0.625 | 64×0.625 | 128×0.6 |
| Pixel spacing  [median (IQR), mm] | 0.672  (0.664, 0.774) | 0.777  (0.742, 0.869) | 0.711  (0.664, 0.810) | 0.711  (0.664, 0.764) | 0.684  (0.684, 0.777) | 0.672  (0.664, 0.774) | 0.711  (0.664, 0.811) |
