# Supplementary material for: Contrast-enhanced CT radiomics for preoperative prediction of stage in epithelial ovarian cancer: a multicenter study
Source: BMC Cancer. 2024 Mar 6;24:307. doi: 10.1186/s12885-024-12037-8 (PMC10916071; doi:10.1186/s12885-024-12037-8)
Supplement: Supplementary file 2 — Supplementary Material 2 [file 12885_2024_12037_MOESM2_ESM.docx]

**S1: Details of radiomics features analysis.**

Data analysis, including data preprocessing, radiomics features selection, building the machine learning models and model evaluation, was perfomed by using the Python Scikit-learn package (Scikit-learn version 1.1.2, <http://scikit-learn.org/>) in Python (version 3.9). The light gradient boosting machine (LightGBM) algorithm used in our article comes from the lightGBM library in Python. The logistic regression, support vector machine, random forest, and decision tree algorithm come from the sklearn library. In addition, a large number of Python ecosystem libraries such as Pandas, Numpy, and Matplotlib were also used in the modeling process.

Statistical tests were performed using R statistical software version 4.2.1. The ROC curves were plotted using the "pROC" package. We used the "rms" package to perform calibration plots construction. LASSO logistic regression was used by "glmnet" package. DCA was performed using the "rmda" package.
