# Supplementary material for: Contrast-enhanced CT radiomics for preoperative prediction of stage in epithelial ovarian cancer: a multicenter study
Source: BMC Cancer. 2024 Mar 6;24:307. doi: 10.1186/s12885-024-12037-8 (PMC10916071; doi:10.1186/s12885-024-12037-8)
Supplement: Supplementary file 3 — Supplementary Material 3 [file 12885_2024_12037_MOESM3_ESM.docx]

**Figure S1**


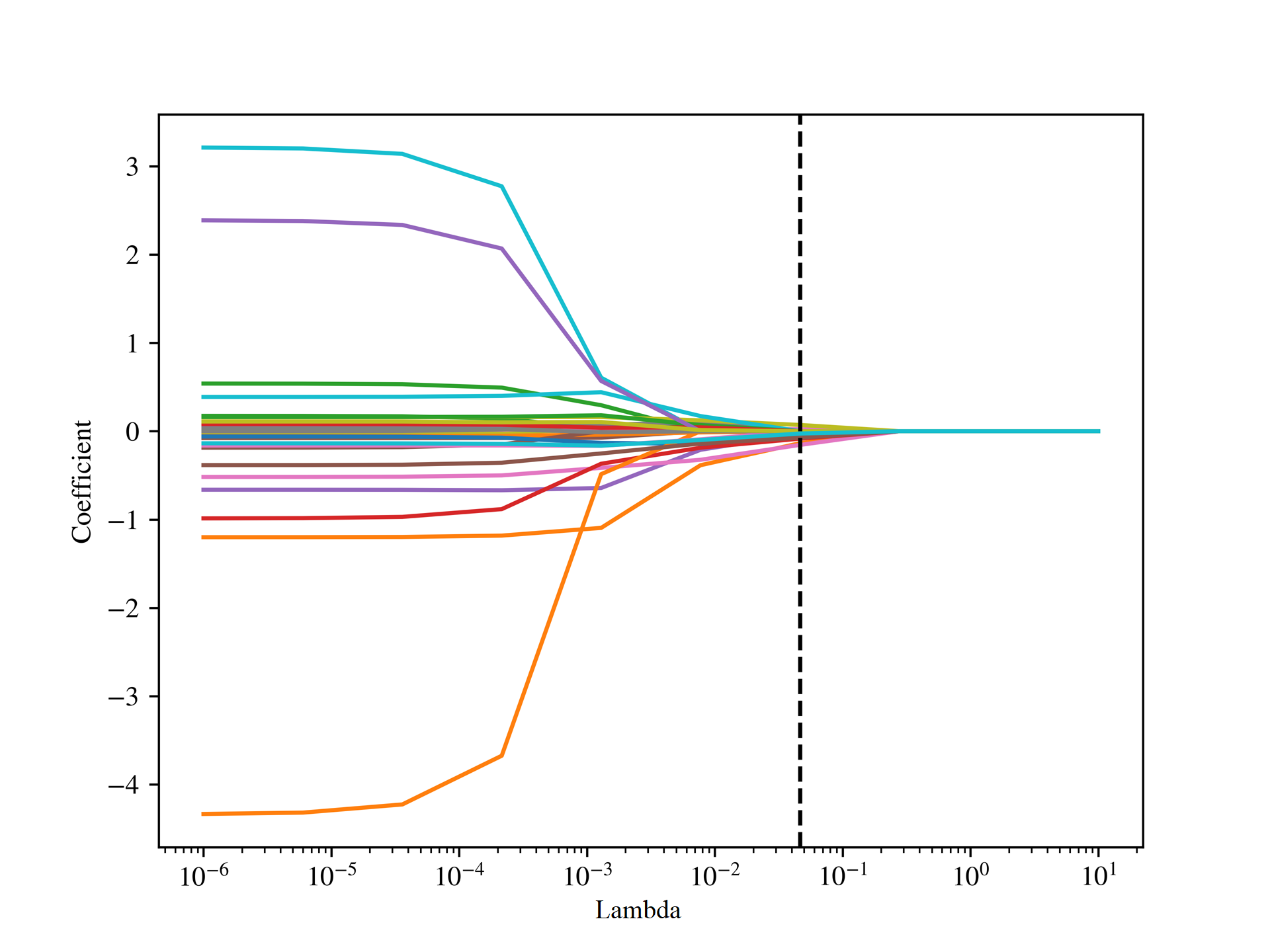


**Figure S1**. Feature selecting to feature selection. The least absolute shrinkage and selection operator (LASSO) algorithm was employed for the purpose of feature selecting. Profile of the LASSO coefficients for radiomics features.
